# The EMA review of trastuzumab emtansine (T-DM1) for the adjuvant treatment of adult patients with HER2-positive early breast cancer

**DOI:** 10.1016/j.esmoop.2021.100074

**Published:** 2021-02-26

**Authors:** J. Delgado, C. Vleminckx, S. Sarac, A. Sosa, J. Bergh, R. Giuliani, H. Enzmann, F. Pignatti

**Affiliations:** 1 Oncology and Haematology Office, European Medicines Agency (EMA), Amsterdam, The Netherlands; 2 Department of Haematology, Hospital Clinic, Barcelona, Spain; 3 Danish Medicines Agency, Copenhagen, Denmark; 4 Committe for Medicinal Products for Human Use (CHMP), EMA, Amsterdam, The Netherlands; 5 Department of Oncology-Pathology, Karolinska Institute and Breast Cancer Centre, Karolinska University Hospital, Stockholm, Sweden; 6 The Clatterbridge Cancer Centre, Liverpool, UK; 7 Bundesinstitut fur Arzneimittel und Medizinprodukte, Bonn, Germany

**Keywords:** trastuzumab emtansine, T-DM1, breast cancer, adjuvant, HER2

## Abstract

Trastuzumab emtansine (T-DM1) is an antibody-drug conjugate of trastuzumab [a monoclonal antibody against human epidermal growth factor receptor 2 (HER2)] and DM1 (an inhibitor of tubulin polymerisation). It was initially approved in the European Union for the treatment of adult patients with HER2-positive unresectable locally advanced or metastatic breast cancer (BC) who had previously received trastuzumab and taxanes. On 18 December 2019, a variation of the marketing authorisation was approved extending this use to the adjuvant therapy of adult patients with HER2-positive early BC who have residual invasive disease in the breast and/or lymph nodes after neoadjuvant taxane-based and HER2-targeted therapy. A phase III randomised, multicentre, open-label trial compared T-DM1 with trastuzumab as adjuvant therapy in patients with HER2-positive early BC who had received preoperative chemotherapy and HER2-targeted therapy followed by surgery, with a finding of invasive residual disease in the breast and/or axillary lymph nodes. The study met its primary endpoint by showing an increased 3-year invasive disease-free survival rate in the T-DM1 arm (88.3%) compared with the trastuzumab arm (77.0%), with an unstratified hazard ratio of 0.50 (95% confidence interval: 0.39-0.64). There was a higher incidence of hepatotoxicity (37.3% versus 10.6%), thrombocytopenia (28.5% versus 2.4%), peripheral neuropathy (32.3% versus 16.9%), haemorrhage (29.2% versus 9.6%) and pulmonary toxicity (2.8% versus 0.8%) in the T-DM1 arm compared with the control arm. The aim of this manuscript was to summarise the scientific review of the application leading to regulatory approval of this additional indication in the European Union.

## Highlights

• T-DM1 was approved for the adjuvant therapy of HER2+ early BC not in pathological complete response after taxanes + anti-HER2 neoadjuvant therapy.
• A phase III randomised trial revealed an increased 3-year invasive disease-free survival rate in patients receiving T-DM1 compared with trastuzumab.
• Patients receiving T-DM1 experienced more hepatotoxicity, thrombocytopenia, neuropathy, bleeding and lung toxicity.

## Introduction

Breast cancer (BC) is the second most common cancer in the world and the most common female cancer, with 2.09 million new cases and approximately 627 000 deaths in 2018 (522 513 new cases and 137 707 deaths in Europe).^1^^,^^2^ Important prognostic and predictive factors in patients with early BC (EBC) are: expression of estrogen/progesterone receptors, human epidermal growth factor receptor 2 (HER2) and proliferation markers (e.g. Ki67); number of involved regional lymph nodes; tumour histology, size and grade; and the presence of peritumoral vascular invasion.^3^

Approximately 10%-20% of tumours overexpress HER2, which is associated with poor clinical outcome, including a 15%-25% risk of recurrence.^4^, ^5^, ^6^, ^7^, ^8^ Locoregional surgery, radiotherapy and systemic therapy (neoadjuvant chemotherapy, HER2-targeted therapy or endocrine therapy) are part of the treatment algorithm for HER2-positive EBC. Patients with HER2-positive tumours >2 cm are recommended to receive neoadjuvant therapy with chemotherapy and trastuzumab, the first-in-class anti-HER2 monoclonal antibody (mAb).^3^^,^^9^ Additionally, pertuzumab, another anti-HER2 mAb, has been approved in combination with trastuzumab and chemotherapy for neoadjuvant (NeoSphere and TRYPHAENA trials) and adjuvant (APHINITY trial) therapy in patients with high-risk HER2-positive EBC^10^; and neratinib was approved for extended adjuvant therapy in patients with HER2-positive EBC in patients who are <1 year from completion of prior adjuvant trastuzumab-based therapy.^11^

Patients who achieve a pathological complete response (pCR), defined as absence of residual invasive cancer on microscopic evaluation of the resected breast and lymph nodes upon completion of the neoadjuvant therapy, have an improved prognosis compared with those with residual invasive disease.^12^^,^^13^ In patients with HER2-positive EBC, a pCR is not achieved in 40%-60% of patients.^14^, ^15^, ^16^, ^17^ Until recently, these patients were recommended to complete 12 months of trastuzumab therapy and expected to have a 3-year disease-free survival (DFS) around 85%-90%.^18^

On 15 November 2013, trastuzumab emtansine (T-DM1, Kadcyla®) was approved in the European Union for the treatment of adult patients with HER2-positive, locally advanced or metastatic BC who had previously received trastuzumab and a taxane, based on a median survival gain of 5.8 months. Since T-DM1 showed activity in patients with progressive disease after chemotherapy plus anti-HER2 therapy in the metastatic setting, it was appropriate to explore its role in patients with HER2-positive EBC who had not had an optimal response to standard neoadjuvant treatment. On 4 February 2019, Roche Registration GmbH applied for an extension of indication for T-DM1 for the adjuvant treatment of adult patients with HER2-positive EBC who had residual disease after neoadjuvant HER2-targeted treatment. The review was conducted by the Committee for Medicinal Products for Human Use (CHMP) and the positive opinion was issued on 14 November 2019.

## Clinical pharmacology

The application to extend the indication of T-DM1 was based on the pivotal study BO27938 (KATHERINE).^19^ During this study, one or more pharmacokinetic (PK) samples were collected from 428 patients in the T-DM1 arm and 405 patients in the trastuzumab arm. A population PK analysis showed that there were no differences in T-DM1 exposure depending on disease status (adjuvant versus metastatic setting). Consistent with PK data from study BO21977 (EMILIA),^20^ repeated dosing of T-DM1 every 3 weeks did not result in any noticeable accumulation of T-DM1 conjugate. Similarly, no difference in serum trastuzumab or plasma DM1 Cmax or Cmin was observed between the KATHERINE and EMILIA studies.

## Study design

This application was based on the primary analysis of the KATHERINE study, a phase III randomised, multicentre, open-label trial comparing T-DM1 versus trastuzumab as adjuvant therapy in patients with HER2-positive EBC who had received preoperative taxane-based chemotherapy and HER2-targeted therapy followed by surgery, with a finding of invasive residual disease in the breast and/or lymph nodes.^19^ Supportive safety data from the phase II study BO22857 were also provided.^21^

T-DM1 was administered intravenously (i.v.) every 3 weeks at the approved dose of 3.6 mg/kg; trastuzumab was administered i.v. every 3 weeks at a maintenance dose of 6 mg/kg after a loading dose of 8 mg/kg. Patients received therapy for 14 cycles but could be prematurely discontinued in case of disease recurrence or unacceptable toxicity. Patients discontinuing T-DM1 could be switched to trastuzumab if appropriate. As neoadjuvant therapy, patients had to have received at least 9 weeks of trastuzumab plus taxane-based chemotherapy. Patients could have also received a second anti-HER2 agent and anthracyclines as part of neoadjuvant therapy. Patients with cardiopulmonary dysfunction were specifically excluded.

The primary endpoint of the study was invasive DFS (IDFS), defined as the time from randomisation to first occurrence of ipsilateral or contralateral invasive breast, locoregional or distant recurrence or death. Secondary endpoints were IDFS including second non-BCs, DFS, overall survival (OS) and distant recurrence-free interval (DRFI).

The sample size of the study was primarily driven by the analysis of IDFS. To detect a hazard ratio (HR) of 0.75 (a 6.5% improvement in 3-year IDFS from 70% to 76.5%), approximately 384 IDFS events were required to achieve 80% power at a two-sided significance level of 5%. Approximately 1484 patients were required, and these 384 events were projected to happen approximately 64 months from first-patient inclusion. With this sample size and 10 years of follow-up, the study had approximately 56% statistical power to detect an HR of 0.8 in OS (a 2.8% improvement in 3-year OS from 85% to 87.8%) at a two-sided significance level of 5%. A hierarchical testing was used to control the overall type I error so that OS was only to be tested if IDFS was statistically significant. Other secondary endpoints were not adjusted for multiplicity.

## Clinical efficacy

**Figure 1:**
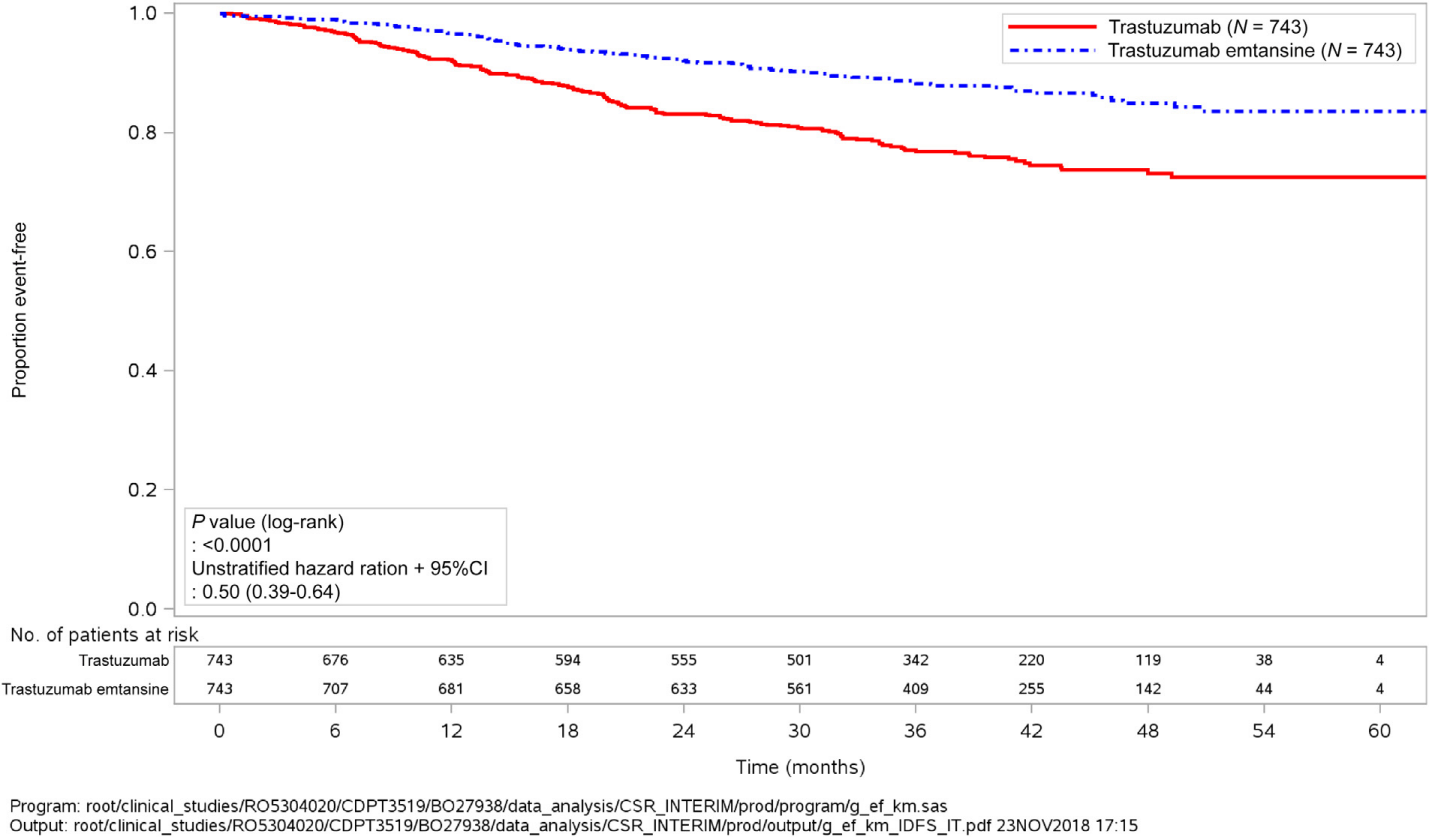
Kaplan–Meier plot of invasive disease-free survival according to randomisation (T-DM1 versus trastuzumab) in the intention-to-treat population (clinical cut-off date: 25 July 2018). CI, confidence interval; T-DM1, trastuzumab emtansine.

A total of 1925 patients with HER2-positive EBC were screened, of whom 1486 were randomised. Almost 20% of patients received two or more anti-HER2 agents as part of their neoadjuvant therapy. The interim analysis of IDFS had been planned to take place whenever ~67% of events (approximately 257 out of 384) had occurred. After 256 events, the clinical cut-off date (CCOD) was set on 25 July 2018, with a median follow-up of 40.9 and 41.4 months for the trastuzumab and T-DM1 arms, respectively. At the CCOD, the study met its primary endpoint with a statistically significant improvement in the 3-year IDFS for T-DM1 over trastuzumab {88.3% versus 77.0%; unstratified HR 0.50 [95% confidence interval (CI): 0.39-0.64]} (Table 1 and Figure 1). As expected, distant recurrence was the most frequent IDFS event in both arms (82.4% and 66.1% of cases in the T-DM1 and trastuzumab arms, respectively). The subgroup analysis of patients who had received two or more anti-HER2 agents before enrolment revealed a similar result [HR 0.54 (95% CI: 0.27-1.06)].

**Table 1:** Favourable and unfavourable effects for trastuzumab emtansine (T-DM1) versus trastuzumab (KATHERINE trial, clinical cut-off date July 2018)

Effect	T-DM1	Trastuzumab	Uncertainties/strength of evidence
Invasive disease-free survival at 3 years, rate (95% CI)	88.27 (85.81-90.72)	77.02 (73.78-80.26)	Unstratified HR 0.50 (0.39-0.64)
Overall survival at 3 years, rate (95% CI)	95.18 (93.58-96.79)	93.59 (91.71-95.47)	Unstratified HR 0.70 (0.47-1.05) *P* = 0.0848 Immaturity of data (6.6% of events)
≥Grade 3 AEs (%)	25.7	15.4	
AEs leading to treatment discontinuation (%)	18.0	2.1	
Thrombocytopenia (%)			
Any grade	28.5	2.4	
Grade ≥3	5.7	0.3	
Haemorrhage (%)			
Any grade	29.2	9.6	
Grade ≥3	0.4	0.3	
Hepatotoxicity (%)			
Any grade	37.3	10.6	
Grade ≥3	1.6	0.4	
Peripheral neuropathy (%)			
Any grade	32.3	16.9	
Grade ≥3	1.6	0.1	
Pulmonary toxicity (%)			
Any grade	2.8	0.8	
Grade ≥3	0.4	0	

AE, adverse event; CI, confidence interval; HR, hazard ratio.

When second non-BCs were added, the 3-year IDFS was 87.68% versus 76.89% for patients receiving T-DM1 and trastuzumab, respectively [unstratified HR 0.51 (95% CI: 0.40-0.66)]. The DFS was 87.41% versus 76.89 for T-DM1 and trastuzumab, respectively [unstratified HR 0.53 (95% CI: 0.41-0.68)]; and the DRFI was also higher for the T-DM1 arm (89.69%) compared with the trastuzumab arm (83.01%) [unstratified HR 0.60 (95% CI: 0.45-0.79)]. Among patients with recurrent disease, 43/91 (47%) in the T-DM1 arm versus 30/165 (18%) in the trastuzumab arm presented central nervous system (CNS) recurrence as the earliest contributing IDFS event. However, the total number of patients with CNS recurrence during follow-up did not differ significantly between arms (45 versus 40 for the T-DM1 and trastuzumab arms, respectively).

Only 98 deaths had occurred compared with the 150 that were estimated at first interim analysis, with a trend towards a prolonged OS for the T-DM1 (unstratified HR 0.70; 95% CI: 0.47-1.05), although data immaturity prevented firm conclusions regarding a non-detrimental effect on OS for patients receiving T-DM1.

## Clinical safety

Safety data from the KATHERINE pivotal study and the phase II study BO22857 were evaluated. Post-marketing signal evaluation from the global safety database had confirmed thrombocytopenia, hepatotoxicity and haemorrhage as T-DM1's major safety risks. By CCOD, 81.0% and 71.4% patients receiving trastuzumab and T-DM1, respectively, had completed therapy. Most patients who prematurely discontinued T-DM1 did so because of AEs: 18% versus 2% in the T-DM1 and trastuzumab arms, respectively. Furthermore, 9.6% patients from the T-DM1 arm ended up being switched to trastuzumab.

Overall exposure to T-DM1 in the KATHERINE trial (median number of cycles = 14) considerably exceeded that from the EMILIA trial (median number of cycles = 9), which granted T-DM1 its indication in the metastatic setting. The proportion of grade ≥3 adverse events (AEs) (26% versus 15%), serious AEs (5% versus 1%) and AEs leading to treatment withdrawal (18% versus 2%) was significantly higher in the T-DM1 arm. Likewise, there was a higher incidence of AEs of special interest: hepatotoxicity (37.3% versus 10.6% all grades; 1.6% versus 0.4% grade ≥3), thrombocytopenia (28.5% versus 2.4% all grades; 5.7% versus 0.3% grade ≥3), peripheral neuropathy (32.3% versus 16.9% all grades; 1.6% versus 0.1% grade ≥3), haemorrhage (29.2% versus 9.6% all grades; 0.4% versus 0.3% grade ≥3) and pulmonary toxicity (2.8% versus 0.8% all grades; 0.4% versus 0% grade ≥3) (Table 1). There was a trend towards a higher incidence of cardiac dysfunction (3.1% versus 5.6%) and asymptomatic decrease in left ventricle ejection fraction (3.1% versus 3.8%) in the trastuzumab arm, but recovery of cardiac function occurred in 80% of the patients without significant differences between the arms.

## Benefit-risk balance

The applicant was seeking an extension of indication of T-DM1 for the adjuvant treatment of adult patients with HER2-positive EBC with invasive residual disease in the breast and/or lymph nodes after neoadjuvant therapy with taxanes plus anti-HER2 targeted therapy, a clinical situation for which there was no specific recommendation at the time.^3^ Although the adjuvant therapy scenario for HER2-positive EBC patients has changed in the last few years, the control arm (trastuzumab) was deemed acceptable considering the period when the study was designed. Allowing patients who discontinued T-DM1 because of toxicity to complete treatment with trastuzumab was endorsed by the CHMP. The primary endpoint of the trial (IDFS) was considered acceptable, even though it did not follow the standardised STEEP definition,^22^ because the possible occurrence of second primary non-BC events was assessed both as a secondary endpoint and served as a sensitivity analysis for the primary endpoint. The remaining exploratory, safety and secondary efficacy endpoints were all considered acceptable. Given the different toxicity profile between both medicinal products, the rationale for not performing a double-blind study was accepted by the CHMP.

The sample size was primarily driven by the analysis of IDFS. The study only had a 56% power to detect a HR of 0.80 in OS, but it was acknowledged that a study adequately powered to show differences in OS may have not been feasible. Still, the applicant presented updated 5-year OS rates (88.1% versus 91.1% for trastuzumab and T-DM1 arms, respectively), further supporting the positive trend observed in the primary analysis. Several sensitivity analyses for IDFS and OS were carried out in which the censoring rules were modified, and the results were concordant with those presented for the primary analysis.

The overall distribution of baseline characteristics, including prior therapy, was balanced between arms. Only 272 patients (18.3%) received trastuzumab + pertuzumab + chemotherapy as neoadjuvant treatment. Although such a combination was not approved when the trial was designed, it currently constitutes the preferred regimen for these patients. The wording of the indication was amended to clearly reflect the studied population (i.e. clarifying the fact that both taxanes and trastuzumab were part of the neoadjuvant scheme). The indication was also amended to reflect that only patients with ‘invasive’ (and not *in situ*) residual disease were included in the trial.

Compared with trastuzumab, T-DM1 demonstrated a clinically meaningful reduction in the overall recurrence rate in the target population. This beneficial effect was primarily observed outside sanctuary sites (distant non-CNS, locoregional and/or contralateral) since there was no difference between arms in CNS recurrence. The results of the trial also suggest that residual invasive disease could also be a predictive biomarker in patients with HER2-positive EBC because patients treated with T-DM1 presented a lower risk of invasive recurrence than those who received trastuzumab.

Significant toxicity was observed in patients treated with T-DM1. Although no new risks were detected, the frequency and severity of known adverse reactions reported with T-DM1 were increased in EBC when compared with advanced disease. Nevertheless, the magnitude of clinical benefit of T-DM1 in the proposed patient population outweighed the observed safety concerns. Measures have been put in place to minimise the risks associated with T-DM1. Health care professionals must be warned about the safety risks derived from thrombocytopenia, haemorrhage and hepatotoxicity. Peripheral neuropathy also deserves attention as it can negatively impact the quality of life.

In conclusion, across subgroups and diverse time-to-relapse endpoints, the overall risk of recurrence in patients with HER2-positive EBC and residual disease after neoadjuvant treatment and surgery was significantly reduced with adjuvant T-DM1 compared with trastuzumab. Given the immature OS data, appropriate follow-up is needed to confirm a non-detrimental effect on OS, and the applicant must submit the final OS analysis from the phase III, randomised, open-label KATHERINE study (due date: 30 June 2024). Based on the review of the submitted data, the CHMP accepted by consensus the variation to the terms of the marketing authorisation, concerning the following change: ‘Extension of indication to include the use of T-DM1 as a single agent for the adjuvant treatment of adult patients with HER2-positive EBC who have invasive residual disease, in the breast and/or lymph nodes, after neoadjuvant taxane-based and HER2-targeted therapy.’

## References

[1] Ferlay J., Colombet M., Soerjomataram I. (2018). Cancer incidence and mortality patterns in Europe: estimates for 40 countries and 25 major cancers in 2018. Eur J Cancer.

[2] Bray F., Ferlay J., Soerjomataram I. (2018). Global cancer statistics 2018: GLOBOCAN estimates of incidence and mortality worldwide for 36 cancers in 185 countries. CA Cancer J Clin.

[3] Cardoso F., Kyriakides S., Ohno S. (2019). Early breast cancer: ESMO Clinical Practice Guidelines for diagnosis, treatment and follow-updagger. Ann Oncol.

[4] Slamon D.J., Clark G.M., Wong S.G. (1987). Human breast cancer: correlation of relapse and survival with amplification of the HER-2/neu oncogene. Science.

[5] Slamon D.J., Godolphin W., Jones L.A. (1989). Studies of the HER-2/neu proto-oncogene in human breast and ovarian cancer. Science.

[6] Allemani C., Sant M., Weir H.K. (2013). Breast cancer survival in the US and Europe: a CONCORD high-resolution study. Int J Cancer.

[7] Lopez-Garcia M.A., Carretero-Barrio I., Perez-Mies B. (2020). Low prevalence of HER2-positive breast carcinomas among screening detected breast cancers. Cancers (Basel).

[8] Cronin K.A., Harlan L.C., Dodd K.W. (2010). Population-based estimate of the prevalence of HER-2 positive breast cancer tumors for early stage patients in the US. Cancer Invest.

[9] Burstein H.J., Curigliano G., Loibl S. (2019). Estimating the benefits of therapy for early-stage breast cancer: the St. Gallen International Consensus Guidelines for the primary therapy of early breast cancer 2019. Ann Oncol.

[10] von Minckwitz G., Procter M., de Azambuja E. (2017). Adjuvant pertuzumab and trastuzumab in early HER2-positive breast cancer. N Engl J Med.

[11] Chan A., Delaloge S., Holmes F.A. (2016). Neratinib after trastuzumab-based adjuvant therapy in patients with HER2-positive breast cancer (ExteNET): a multicentre, randomised, double-blind, placebo-controlled, phase 3 trial. Lancet Oncol.

[12] Rastogi P., Anderson S.J., Bear H.D. (2008). Preoperative chemotherapy: updates of National Surgical Adjuvant Breast and Bowel Project Protocols B-18 and B-27. J Clin Oncol.

[13] Broglio K.R., Quintana M., Foster M. (2016). Association of pathologic complete response to neoadjuvant therapy in HER2-positive breast cancer with long-term outcomes: a meta-analysis. JAMA Oncol.

[14] Buzdar A.U., Ibrahim N.K., Francis D. (2005). Significantly higher pathologic complete remission rate after neoadjuvant therapy with trastuzumab, paclitaxel, and epirubicin chemotherapy: results of a randomized trial in human epidermal growth factor receptor 2-positive operable breast cancer. J Clin Oncol.

[15] Gianni L., Eiermann W., Semiglazov V. (2010). Neoadjuvant chemotherapy with trastuzumab followed by adjuvant trastuzumab versus neoadjuvant chemotherapy alone, in patients with HER2-positive locally advanced breast cancer (the NOAH trial): a randomised controlled superiority trial with a parallel HER2-negative cohort. Lancet.

[16] Cortazar P., Zhang L., Untch M. (2014). Pathological complete response and long-term clinical benefit in breast cancer: the CTNeoBC pooled analysis. Lancet.

[17] Loibl S., Jackisch C., Lederer B. (2015). Outcome after neoadjuvant chemotherapy in young breast cancer patients: a pooled analysis of individual patient data from eight prospectively randomized controlled trials. Breast Cancer Res Treat.

[18] Piccart-Gebhart M.J., Procter M., Leyland-Jones B. (2005). Trastuzumab after adjuvant chemotherapy in HER2-positive breast cancer. N Engl J Med.

[19] von Minckwitz G., Huang C.S., Mano M.S. (2019). Trastuzumab emtansine for residual invasive HER2-positive breast cancer. N Engl J Med.

[20] Verma S., Miles D., Gianni L. (2012). Trastuzumab emtansine for HER2-positive advanced breast cancer. N Engl J Med.

[21] Krop I.E., Suter T.M., Dang C.T. (2015). Feasibility and cardiac safety of trastuzumab emtansine after anthracycline-based chemotherapy as (neo)adjuvant therapy for human epidermal growth factor receptor 2-positive early-stage breast cancer. J Clin Oncol.

[22] Hudis C.A., Barlow W.E., Costantino J.P. (2007). Proposal for standardized definitions for efficacy end points in adjuvant breast cancer trials: the STEEP system. J Clin Oncol.

